# The γδ T Lymphocytes of the Perinatal Murine Thymus

**DOI:** 10.1155/1995/57536

**Published:** 1995

**Authors:** Michelle I. Zorbas, Roland Scollay

**Affiliations:** 1 The Walter and Eliza Hall Institute of Medical Research Post Office Royal Melbourne Hospital Victoria 3050 Australia; 2 The Centenary Institute of Cancer Medicine and Cell Biology Locked Bag No. 6 Newtown NSW 2042 Australia; 3 INSERM U345 CHU Necker 156 rue de Vaugirard Paris Cedex 15 75730 France

**Keywords:** γδ lymphocytes, heat-stable antigen, lymphocyte development, mouse thymus, Thy-1

## Abstract

We have previously shown that the adult thymus contains three subsets of γδ T cells that can be defined by the expression of Thy-1 and heat-stable antigen (HSA). In this study, the number of cells in each of these thymic γδ populations was investigated at different stages throughout life. In adult mice, the populations stayed relatively constant, however, in contrast, there were major variations in them early in development. It was shown that only two of the γδ populations were present in the prenatal thymus, a major population of Thy-1^+^ HSA^-^ cells, and a smaller population of Thy-1^+^ HSA^-^ cells. However, after birth, most of the Thy-1^+^ HSA^-^cells appear to loose the Thy-1 antigen, forming the third population of HSA^-^ Thy-1^-^cells. The adult configuration of populations appeared to be established within the first week after birth. Therefore, whereas the γδ populations stayed relatively constant from this time point onwards, there were major variations early in development. Throughout life, most γδ thymocytes are CD4^-^ CD8^-^, however, in the neonatal thymus, there are some CD4^+^ and CD8^+^ γδ thymocytes, and these are contained in the Thy-1^+^ HSA^-^ population.


*Developmental Immunology*, 1995, Vol 4, pp. 93–100
Reprints available directly from the publisher
Photocopying permitted by license only

© 1995 Harwood Academic Publishers GmbH
Printed in Singapore

### The $\gamma\delta$ T Lymphocytes of the Perinatal Murine Thymus

MICHELLE I. ZORBAS*† and ROLAND SCOLLAY‡†

†*The Walter and Eliza Hall Institute of Medical Research, Post Office Royal Melbourne Hospital, Victoria 3050, Australia*
‡*The Centenary Institute of Cancer Medicine and Cell Biology, Locked Bag No. 6, Newtown, NSW 2042, Australia*

We have previously shown that the adult thymus contains three subsets of $\gamma\delta$ T cells that can be defined by the expression of Thy-1 and heat-stable antigen (HSA). In this study, the number of cells in each of these thymic $\gamma\delta$ populations was investigated at different stages throughout life. In adult mice, the populations stayed relatively constant, however, in contrast, there were major variations in them early in development. It was shown that only two of the $\gamma\delta$ populations were present in the prenatal thymus, a major population of Thy-1$^+$ HSA$^-$ cells, and a smaller population of Thy-1$^+$ HSA$^+$ cells. However, after birth, most of the Thy-1$^+$ HSA$^-$ cells appear to loose the Thy-1 antigen, forming the third population of HSA$^-$ Thy-1$^-$ cells. The adult configuration of populations appeared to be established within the first week after birth. Therefore, whereas the $\gamma\delta$ populations stayed relatively constant from this time point onwards, there were major variations early in development. Throughout life, most $\gamma\delta$ thymocytes are CD4$^-$ CD8$^-$, however, in the neonatal thymus, there are some CD4$^+$ and CD8$^+$ $\gamma\delta$ thymocytes, and these are contained in the Thy-1$^+$ HSA$^-$ population.

KEYWORDS: $\gamma\delta$ lymphocytes, heat-stable antigen, lymphocyte development, mouse thymus, Thy-1.

### INTRODUCTION

The thymus is the major site for development of both $\alpha\beta$ and $\gamma\delta$ T cells. The different stages of $\alpha\beta$ T-cell development have been well described, with the cells progressing from an early CD4$^{lo}$ stage, through double negative (CD4$^-$ CD8$^-$) and double-positive (CD4$^+$ CD8$^+$) stages, and finally maturing into the single-positive thymocytes that seed the periphery (Nikolic-Zugic, 1991; Rothenberg, 1992; Shortman, 1992). In contrast, the process of $\gamma\delta$ T-cell development is largely unknown. It is clear that there are early waves of $\gamma\delta$ cells produced in the thymus during ontogeny, each with characteristic T-cell receptor (TCR) gene expression and tissue homing ability (Havran and Allison, 1988; Houlden et al., 1988; Ito et al., 1989; Itohara et al., 1990; Havran et al., 1991), and it has been shown that $\gamma\delta$ T cells migrate from the adult thymus to the spleen and lymph nodes (Kelly et al., 1993). However, some $\gamma\delta$ populations, including those in the intestinal epithelium, appear to be produced extrathymically (De Geus et al., 1990; Bandeira et al., 1991; Guy Grand et al., 1991). Thus, there seem to be both intrathymic and extrathymic developmental pathways for $\gamma\delta$ T cells.

We have previously described the double-negative $\gamma\delta$ cells found within the adult thymus. They consist of three populations that can be defined on the basis of HSA and Thy-1 expression and that further differ in their V$\gamma$ gene usage, surface-marker expression levels, and population turnover rates within the thymus (Zorbas and Scollay, 1993). The three populations comprise 52% (HSA$^+$ Thy-1$^+$), 14% (HSA$^-$ Thy-1$^+$) and 31% (HSA$^-$ Thy-1$^-$) of the double-negative $\gamma\delta$ T cells in CBA mice. It appeared that only one of the subsets, that expressing both HSA and Thy-1, was the source of the $\gamma\delta$ T cells that seeded the periphery. This was suggested by the similarities in Thy-1, HSA, Mel-14, and Pgp-1 expression, the V$\gamma$ gene usage between this population and the recent $\gamma\delta$ emigrants, and because the turnover of only this thymic population was sufficient to account for the number of cells migrating from the thymus.

In order to determine when these populations of $\gamma\delta$ cells first appeared in the thymus, whether they were present throughout life and whether any of

*Corresponding author, present address: INSERM U345 CHU Necker, 156 rue de Vaugirard, 75730 Paris Cedex 15, France.

the γδ T-cell populations accumulated with age, a study of the relative proportions of the thymic γδ T-cell populations during ontogeny was carried out. From this work, it became apparent that there were changes early in development and these have been further investigated in terms of the Thy-1, HSA, and CD4 and CD8 expression. This study reveals the rapid changes that give rise to the adult conformation within the first few days following parturition and the steady state of these populations throughout adult life.

### RESULTS

### Changes in the Thymic γδ Populations

The number of total γδ T cells was determined in the thymus from mice of various ages from birth to 200 days and compared with total thymocyte number. The number of γδ T cells increased up to 5 weeks of age and then gradually declined and appeared to stay proportional to the total number of thymocytes (Fig. 1A). Most γδ thymocytes are $CD4^-$ $CD8^-$ (double-negative), and among these cells there are three main populations. Figure 1B shows the number of cells within each of these populations over the same time scale as before. It can be seen that the relative proportions of these populations was also constant throughout life.

When the γδ populations were analyzed during the first few weeks of life, it was seen that there were significant changes in their proportions during this early phase of development (Fig. 1C). The $HSA^-$ $Thy\text{-}1^-$ population, initially the smallest population, increased rapidly to become one of the two major γδ populations by day 10. There were also increases in the other two populations at this time, most notably the $HSA^+$ $Thy\text{-}1^+$ population. Therefore, it appeared that very little variation in the γδ thymocyte populations occurs throughout development, except during the first week after birth.

### The Perinatal γδ Populations

To investigate the early changes in the γδ populations, a study of late fetal to early neonatal γδ thymocytes was undertaken. The HSA and Thy-1 expression of the γδ cells, as well as CD4 and CD8 expression, were included in this study. Figure 2 contains representative contour plots of the HSA and Thy-1 fluorescence of the γδ populations

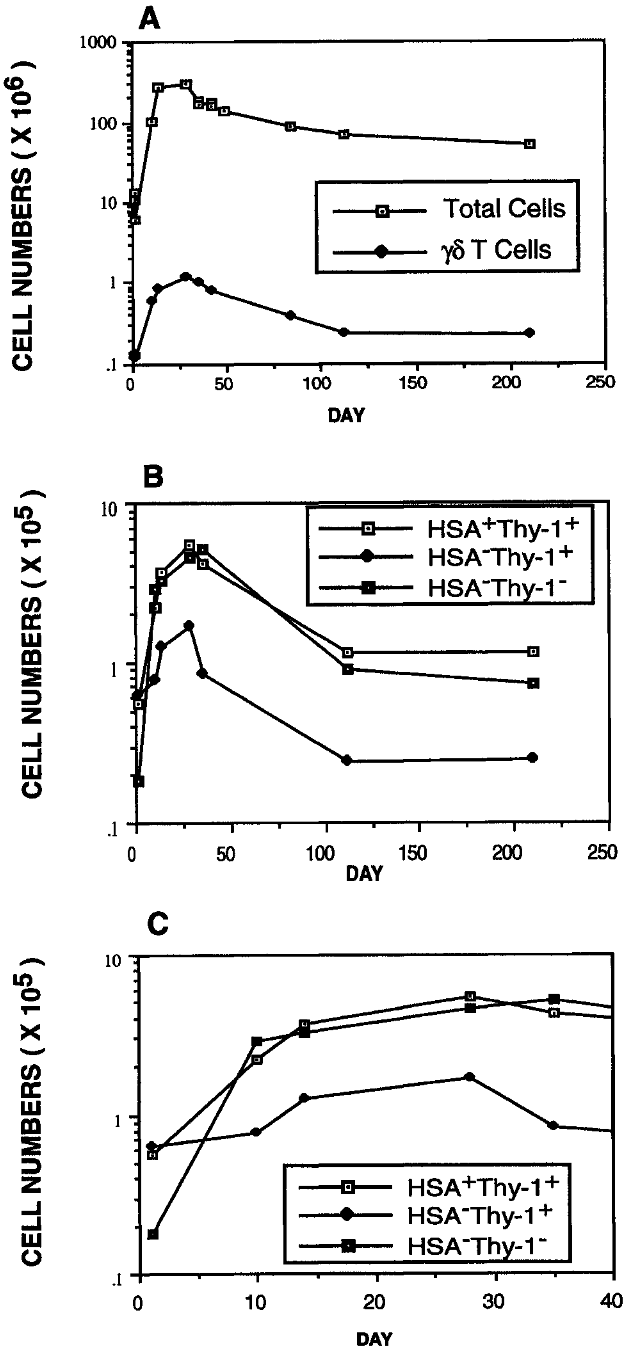

FIGURE 1. The changes in the γδ populations that occur during thymic development. (A) The numbers of γδ thymocytes and total thymocytes are plotted on a logarithmic scale over time in days. Cell numbers are derived from counts of thymocyte suspensions and electronic gating of γδ $TCR^+$ cells from thymuses of 6 to 20 mice at each time point. The γδ T-cell population comprises 0.5 to 1% of total thymus number throughout life. (B) The number of γδ T cells within each of the three populations defined by HSA and Thy-1 are given over the same time scale as in A. (C) Detail of the population changes during the first 5 weeks following birth. The changes in the proportions of the populations in the first week can be seen.

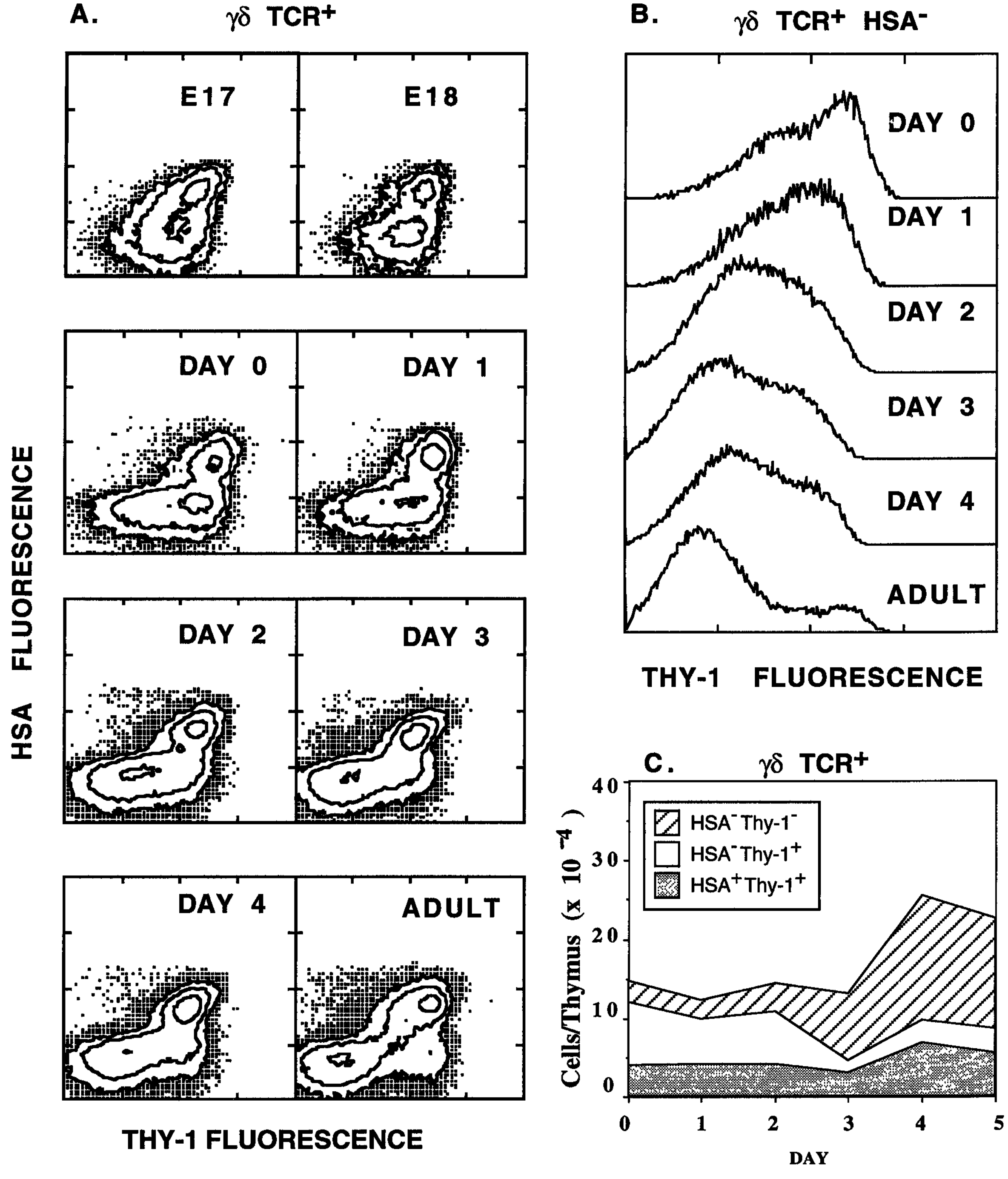

FIGURE 2. The perinatal γδ populations in the thymus. Flow cytometric analysis was performed on $CD4^-$ $CD8^-$ cells isolated from the thymuses of 6 to 20 mice, depending on the age of the mice and thymus size. The data shown are representative of three experiments. (A) Contour plots of the HSA and Thy-1 fluorescence of total γδ T thymocytes from mice at E17 to 4 days after birth. The populations begin to resemble the adult distribution by day 4 and are identical by 2 weeks (data not shown). (B) In order to clearly identify the changes in the Thy-1 expression over this time period, the files shown in the contour plots were electronically gated $HSA^-$ and γδ $TCR^+$. The histograms show the Thy-1 expression on all the $HSA^-$ cells and the shift in the level that occurs at days 1 to 3 is apparent. (C) Graph of the actual numbers of cells present within each γδ T-cell subset during the first 5 days postnatal. Cell numbers are given on a linear scale and are represented by the differently shaded areas. Total γδ thymocytes are therefore the combined total of the three areas. The shift in Thy-1 expression that occurs at day 2 to 3 can been seen in the loss of $HSA^-$ $Thy\text{-}1^+$ cells and an increase of a similar number of cells in the $HSA^-$ $Thy\text{-}1^-$ population.

in $CD4^-$ $CD8^-$ thymus from E17 through to day 4 of development. The earliest γδ cells were mostly Thy-$1^+$ $HSA^-$ or Thy-$1^+$ $HSA^+$. Over the following few days, a Thy-$1^-$ $HSA^-$ population arose. Figure 2B contains histograms of the Thy-1 expression of the $HSA^-$ cells on each day. There appeared to be a gradual loss of Thy-1 and it was possible that the Thy-$1^+$$HSA^-$ cells were giving rise to the Thy-1 HSA cells at day 2 to 3 postnatal by the loss of surface Thy-1.

Figure 2C is an area plot of the actual cell numbers within each γδ population from birth to day 5 with the three shaded areas representing the cell number within each population. When plotted in this manner, the top line represents the total γδ cell numbers in the thymus and relative changes can be seen. At 2–3 days after birth, there was an increase in the $HSA^-$ Thy-$1^-$ population and a corresponding decrease in the number of $HSA^-$ Thy-$1^+$ cells. The $HSA^-$ Thy-$1^+$ population was at it highest level relative to the other γδ populations at this point, and after this declined to be the smallest thymic γδ population and remained at this low proportion throughout life. During this time, the $HSA^+$ Thy-$1^+$ population remained relatively constant, and then began to increase in number toward the peak at 5 to 6 weeks.

Previous reports have shown that few γδ cells express CD4 or CD8 in the adult thymus, although a significant number of cells do before birth (Fisher and Ceredig, 1991). There is a peak of $CD8^+$ cells at E18, which then declines, and from this point, the proportion of double-negative cells increases (Penit and Vasseur, 1989). It is not known how the γδ cells bearing surface $CD4^+$ or $CD8^+$ relate to the double-negative γδ cells or if they develop in a double-negative to double-positive pathway as do αβ T cells. Therefore, the HSA and Thy-1 expression of the double-negative and CD4/8-expressing γδ populations were compared. As this analysis required γδ TCR staining, HSA and Thy-1, the CD4 and CD8 molecules were stained in the same fluorescence channel. Therefore, the double-positive and single-positive cells cannot be distinguished in these experiments, although it is known that in the newborn, most of the γδ cells that are not double-negative are $CD8^+$ (Fisher and Ceredig, 1991).

The histograms in Fig. 3A show the CD4/8 fluorescence of gated γδ T cells compared to total thymus for days 0 to 4 of postnatal development. The major proportion of the total thymocytes at all these time points is $CD4^+CD8^+$. Few γδ T cells fall within the region for $CD4^+8^+$ cells in any of the plots, although day 2 has the largest proportion of positive cells. The positive cells never form a discrete peak, but appear to tail out from the "negative" cells. In order to look at the γδ cells with the highest levels of these markers, an arbitrary electronic gate was set at fluorescence level 25 on the log scale. The level of CD4/8 negative staining can be seen in Fig. 3C on the $HSA^+$γδ populations and it is predominantly below fluorescence level 25.

The HSA and Thy-1 fluorescence of these gated CD4/$8^+$ γδ T cells are shown in contour plots in Fig. 3B at each day of assay and the expression levels can be directly compared to the plots in Fig. 2A. These $CD4^+/8^+$ γδ cells are mostly Thy-$1^+$and $HSA^-$ with the Thy-1 fluorescence levels being similar to the Thy-1 fluorescence of the major proportion of γδ cells at each time point. As some CD4/$8^-$ cells may be included in the gated population, the CD4/8 fluorescence versus the HSA flourescence is also given (Fig. 3C). The data again show that during the first five days of life, the cells brightest for CD4/8 are $HSA^-$. In the adult (the top plots ), the CD4/$8^+$ cells are very rare but do appear to be mainly $HSA^+$, as indicated in Fig. 3B. It has been previously reported that a small proportion of adult thymic γδ cells are not double-negative, but express CD8, and an even smaller number are $CD4^+$ $CD8^+$(Fisher and Ceredig, 1991).

We have previously reported that in the adult, there is a difference in the level of TCR expressed by the different γδ subsets, with the Thy-$1^-$ $HSA^-$ population having a discrete peak with high-level staining. This is less obvious in the newborn or postnatal mice, where all three populations have bright TCR staining, and at day 0, the Thy-$1^-$ $HSA^-$ and Thy-$1^+$ $HSA^-$ TCR fluorescence levels are identical (data not shown), perhaps indicating fewer differences between these cells at this early point in development.

### DISCUSSION

Within the adult thymus, the $γδ^+$ lymphocytes are heterogeneous by a number of criteria that may indicate different past history, homing, or migratory potential of the subpopulations. The data presented here indicate that the γδ populations early in development are also complex and may play different roles in the thymus and elsewhere in the organism.

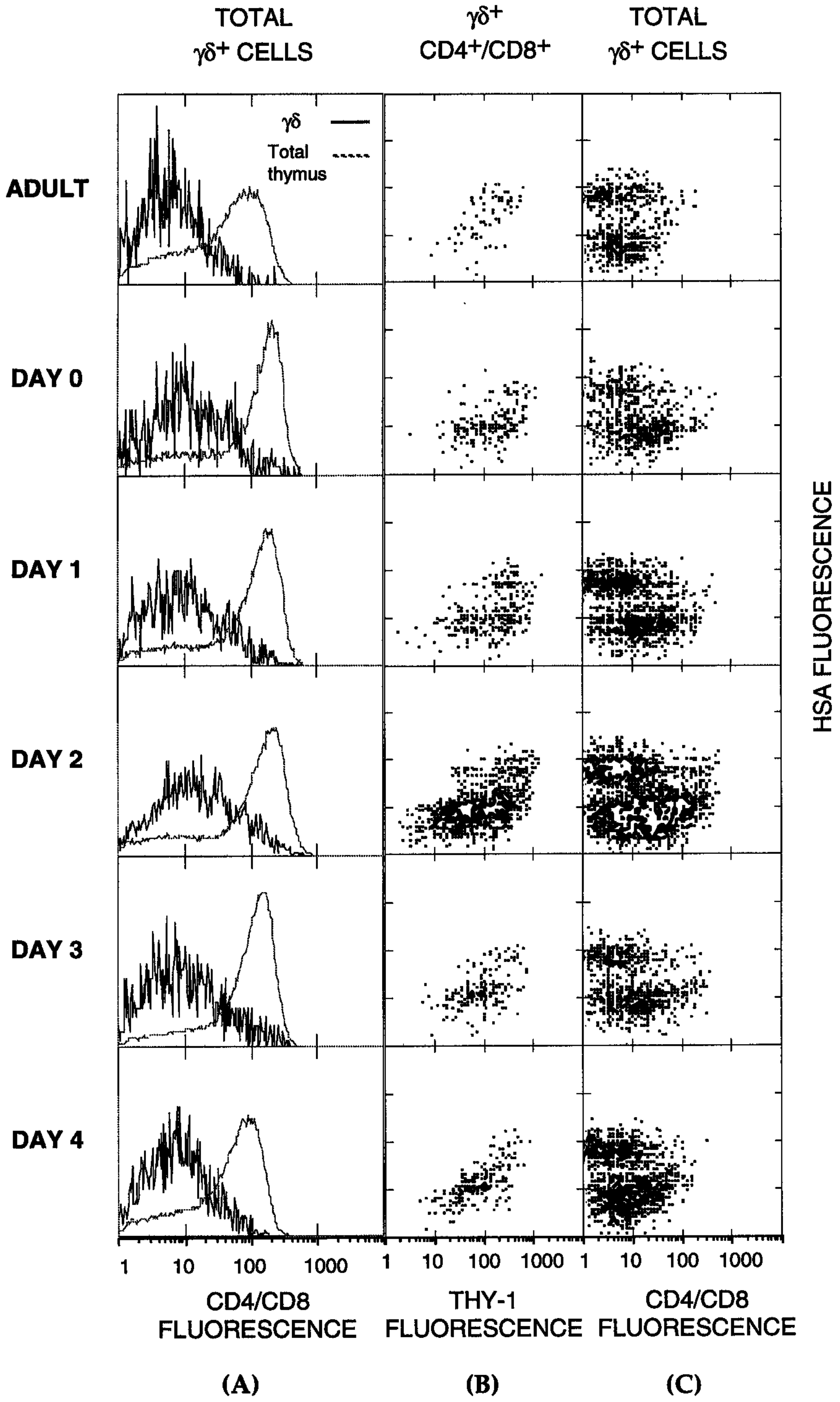

FIGURE 3. The Thy-1 and HSA expression of the γδ thymocytes that bear CD4 or CD8. The analysis was performed on cells isolated from the thymuses of 6 to 20 mice at ages 0 to 4 days and adult. The data shown are representative of three experiments. (A) The histograms show the CD4/8 fluorescence of total γδ T cells (solid line) for the first 5 days following birth and in the adult thymus. The CD4/8 profile of total thymus at each time point is included to indicate the level of positive staining (dotted line). (B) The HSA and Thy-1 profiles of the γδ T thymocytes that have a CD4/8 fluorescence above an arbitrary level of fluorescence 25 on the log scale. (C) Contour plots of gated γδ $TCR^+$ cells. The CD4/8 fluorescence is plotted against the HSA fluorescence to demonstrate that the majority of $CD4/8^+$ cells have an $HSA^-$ phenotye as indicated in (B).

This study has revealed that the $\gamma\delta$ populations were in a steady state in the adult thymus, with significant changes occurring only early in development. This early phase corresponds with a stage when the $\gamma\delta$ cells are making a major contribution to the peripheral lymphocyte populations. The major populations in the perinatal mouse thymus were the HSA$^-$ Thy-1$^+$ cells and the HSA$^+$ Thy-1$^+$ cells. The HSA$^-$ Thy-1$^-$ cells that were numerous in the adult may arise from the HSA$^-$ Thy-1$^+$ cells and then remain stagnant; as we have previously shown, they are turning over extremely slowly in the adult thymus (Zorbas and Scollay, 1993). It is interesting that in the adult, it was these HSA$^-$Thy-1$^-$cells that expressed the brightest level of $\gamma\delta$ T-cell receptor on the surface, suggesting this population may be postselection or at a more mature developmental stage than the other thymic $\gamma\delta$ cells. In the fetus, however, this difference in T-cell receptor level was not apparent, and all three populations had bright $\gamma\delta$ TCR fluorescence. The striking changes seen in the pattern of the $\gamma\delta$ populations that were present during development also highlights the fact that the role of the $\gamma\delta$ T cells may be quite different in the young mouse compared to the adult.

The data presented also demonstrated that although the rare adult $\gamma\delta$ cells that expressed CD8 or CD4 were mainly Thy-1$^+$ HSA$^+$, in the neonate, this population was clearly Thy-1$^+$ HSA$^-$. In each case, this represented the largest population of $\gamma\delta$ cells. In the adult, this was also the only $\gamma\delta$ population that was dividing, whereas in the newborn mouse, most $\gamma\delta$ cells are dividing (Ceredig, 1990). So the CD4/8$^+$$\gamma\delta$ cells seem to be within the major population both in the early mouse and the adult. Indeed, the unimodal staining pattern may indicate that many cells express low levels of CD8 or 4, and that the positive cells are simply the upper end of a normal distribution.

It has been clear for many years that the waves of early $\gamma\delta$ populations make a major contribution to the peripheral lymphoid pool during fetal life. Therefore, it is not surprising that the $\gamma\delta$ populations defined by HSA and Thy-1 reflect the rapid changes in the thymic $\gamma\delta$ populations that occur in the perinatal period. The developmental potential of fetal stem cells also appears to be very different to that of adult stem cells (Ikuta et al., 1990; Ogimoto et al., 1990), and it is likely that up to 1 week of age in the mouse, the thymocytes and peripheral T cells are derived from the initial stem-cell seeding (Jotereau et al., 1987). Therefore, differences in the thymus between the neonatal and adult mouse would not be unexpected; for instance, $\alpha\beta$ T-cell development is accelerated in the fetal mouse when compared to the adult.

From this study, there also appear to be major differences in the $\gamma\delta$ T cells between the adult and the neonate. In the perinatal thymus, the major population of $\gamma\delta$ cells is Thy-1$^+$ HSA$^-$, and so it is possible that many of these cells emigrate to the periphery and this may also include the CD8$^+$ $\gamma\delta$ thymocytes that are thought to seed the gut around this time (Havran and Allison, 1988). In the adult, the population that gives rise to emigrants is Thy-1$^+$ HSA$^+$ and there are fewer Thy-1$^+$ HSA$^-$ cells in the thymus. In addition, these cells are not dividing and not turning over in the adult thymus (Zorbas and Scollay, 1993). This means they may be remnants from fetal development. We have previously shown they express mainly $V_{\gamma 1}$ and $V_{\gamma 2}$ genes, which are common in the late fetal and neonatal thymus. They do not appear to result from very early events in ontogeny as they express little $V_{\gamma 3}$ or $V_{\gamma 4}$, which predominate at that stage.

In the neonate, it appears that the Thy-1$^+$ HSA$^-$ cells may mature to form the Thy-1$^-$ HSA$^-$ population, which raises the question of whether this also occurs in the adult.

This would be similar to $\alpha\beta$ development where the cells lose Thy-1 and HSA as they mature. However, whereas these cells are dividing in the neonate, in the adult, the turnover rate of these two populations is only about 1% per day (Zorbas and Scollay, 1993). Therefore, this would be a very slow process. However, because the turnover rate of these two populations is so similar, the Thy-1$^+$ HSA$^-$ cells may contribute to the Thy-1$^-$ HSA$^-$ cells.

The turnover and cell numbers in the fetal thymus have been reported (Penit and Vasseur, 1989). The thymocyte numbers do not increase between E18 and 3 days postnatal, after which there is rapid expansion. This steady total cell number and then growth beginning at day 3 are also reflected in the $\gamma\delta$ populations present during this time period. It is interesting that the first appearance of the HSA$^-$ Thy-1$^-$ $\gamma\delta$ T cells at 3 days postnatal corresponds with the onset of rapid thymocyte proliferation. It may be that these cells have their major role at this time in development, perhaps influencing $\alpha\beta$ cell development, as they seem largely inactive in the adult. Transgenic mice lacking $\gamma\delta$ T cells still demonstrate normal $\alpha\beta$ T-cell development (Itohara et al., 1993), however, it may be that in these mice, another cell type can substitute for the role of the γδ T cells at this stage.

In summary, the experiments reported here have shown marked and rapid changes in the γδ populations around the time of birth. The data show that there is a complex series of events occurring during the change from fetal subsets ($V_\gamma 3^+$ and $V_\gamma 4^+$) to the adult pattern, which appears 1–2 weeks after birth. This may reflect the changing and varied roles of γδ cells in the perinatal mouse and the adult.

### MATERIALS AND METHODS

The mice used for these experiments were CBA/CaH Wehi strain, bred at the Walter and Eliza Hall Institute animal facility under specific pathogen-free conditions. Adult mice were 5 to 6 weeks old and other mice were used at the ages indicated. Day 0 is within 12 hours of birth and day 1 within 36 hours.

Adult animals were sacrificed using $CO_2$ inhalation, and freshly taken organs were placed into ice-cold balanced salt solution with 2% fetal calf serum (BSS/FCS) (Shortman et al., 1972). Fetal and neonatal mice were killed on ice and the thymus was dissected out under a binocular microscope and treated as before, although keeping the volume of media minimal. The thumus was disaggregated through wire mesh and washed once by resuspension in BSS/FCS with a 1-ml FCS underlay, and centrifugation at 1700 rpm for 7 mins. Red blood cells were removed from fetal thymus with red blood cell removal buffer (Shortman et al., 1972) as the cells cannot be excluded on the basis of size. Cell counts were performed by eosin exclusion with a hemocytometer, with duplicate counts of at least 100 cells each.

To enrich the $CD4^-$ $CD8^-$cells from adult thymus, complement mediated killing using anti-CD8 (49.11.1, Hogarth et al., 1982) and anti-CD4 (172.4, Ceredig et al., 1985) was performed as described by Wilson et al (1988). The cells were then loaded onto a density gradient to recover viable cells (Scollay and Shortman, 1983). The neonatal mice have increased proportions of double-negative cells and this enrichment step was not used. Instead, the γδ cells were electronically gated for further analysis.

Cell staining was carried out in 2-ml siliconized glass conical tubes, as previously described (Wilson et al., 1988). Ten μl of antibody was used for each $10^6$cells with 30 μl the minimum volume. All antibodies were titrated to determine the optimum concentration for use. Cells were resuspended by flicking in the appropriate dilution of antibody and then incubated on ice for 20 min. Cells were washed in BSS/FCS with a 0.25-ml underlay of FCS and were pelleted by centrifugation as described. The staining protocol was anti-CD8 (53.6.7; Ledbetter and Herzenberg, 1979) and anti-CD4 (GK1.5; Dialynas et al., 1983), which were detected using anti-Ig-APC (Caltag). The anti-Ig was then blocked with rat/mouse Ig, and this was competed against the antibodies to the γδ TCR (GL31A-FITC; Goodman and Lefrancois, 1989), HSA (M169-PE; Springer et al., 1978), and Thy-1.2 (30H12-Biotin; Ledbetter and Herzenberg, 1979), and the final stage was Avidin-Texas Red (Amersham).

Five-color flow cytometric analysis was performed using a $\text{FACStar}^{\text{Plus}}$ or modified Facs II (Battye, 1985) (supplied by Becton Dickinson, Sunnyvale, CA). Populations were electronically gated and cell numbers derived as a proportion of total thymocyte numbers. Files of 100,000 events were collected where possible; gated files are subsets of these where nonviable cells or particulate matter was excluded on the basis of forward and side scatter and propidium iodide was used to exclude dead cells. The data are shown as histograms or contour plots with the lowest level indicated by dots.

The γδ $V_\gamma$ gene nomenclature used throughout is that of Garman et al. (1986).

### ACKNOWLEDGMENTS

The authors would like to thank F. Battye, R. Rossie, and R. Muir for excellent technical help with flow cytometric analysis and K. Shortman and K. Lundberg for critical review of the manuscript. MZ was supported by an Australian Postgraduate Research Award and the research was funded by the National Health and Medical Research Council of Australia.

*(Received May 20, 1994)*

*(Accepted August 24, 1994)*

### REFERENCES

Bandeira A., Itohara S., Bonneville M., Burlen-Defranoux O., Mota-Santos T., Coutinho A., and Tonegawa S. (1991). Extrathymic origin of intestinal intraepithelial lymphocytes bearing T-cell antigen receptor γδ. Proc. Natl. Acad. Sci. USA **88:** 43–47.

Battye F.L. (1985). A FACS II with dual lasers and five sort parameters. In Flow Cytometry: Instrumentation and data analysis, van Dilla M.A., Melamed M.R., Laerum O.D., Dean P.N., Eds. (:) pp. 228–231.

Ceredig R. (1990). Intrathymic proliferation of perinatal mouse alpha beta and gamma delta T cell receptor-expressing mature T cells. Int. Immunol. **2(9):** 859–867.

Ceredig R., Lowenthal J.W., Nabholz M., and MacDonald H.R. (1985). Expression of interleukin-2 receptors as a differentiation marker on intrathymic stem cells. Nature **314(6006):** 98–100.

De Geus B., van den Enden M., Coolen C. Nagelkerken L., van der Heijden P., and Rozing J. (1990). Phenotype of intraepithelial lymphocytes in euthymic and athymic mice: implications for differentiation of cells bearing a CD3-associated gamma delta T cell receptor. Eur. J. Immunol. **20(2):** 291–298.

Dialynas D.P., Quan Z.S., Wall K.A., Pierres A., Quintans J., Loken M.R., Pierres M., and Fitch F.W. (1983). Characterization of the murine T cell surface molecule, designated L3T4, identified by monoclonal antibody GK1.5: Similarity of L3T4 to the human Leu-3/T4 molecule. J. Immunol. **131(5):** 2445–2451.

Fisher A.G., and Ceredig R. (1991). Gamma delta T cells expressing CD8 or $CD4^{low}$ appear early in murine fetal thymus development. Int. Immunol. **3(12):** 1323–1328.

Garman R.D., Doherty P.J., and Raulet D.H. (1986). Diversity, rearrangement and expression of murine T cell gamma genes. Cell **45:** 733–742.

Goodman T., and Lefrancois L. (1989). Intraepithelial lymphocytes. Anatomical site, not T cell receptor form, dictates phenotype and function. J. Exp. Med. **170(5):** 1569–1581.

Guy Grand D., Cerf Bensussan N., Malissen B., Malassis Seris M. Briottet C., and Vassalli P. (1991). Two gut intraepithelial $CD8^+$ lymphocyte populations with different T cell receptors: A role for the gut epithelium in T cell differentiation. J. Exp. Med. **173(2):** 471–481.

Havran W.L., and Allison J.P. (1988). Developmentally ordered appearance of thymocytes expressing different T-cell receptors. Nature **335:** 443.

Havran W.L., Carbone A., and Allison, J.P. (1991). Murine T cells with invarient $\gamma\delta$ antigen receptors: origin, repertoire, and specificity. Sem. Immunol. **3:** 89–97.

Hogarth P.M., Edwards J., McKenzie I.F., Goding J.W., and Liew F.Y. (1982). Monoclonal antibodies to the murine Ly-2.1 cell surface antigen. Immunol. **46(1):** 135–144.

Houlden B.A., Cron R.Q., Coligan J.E., and Bluestone J.A. (1988). Systematic development of distinct T cell receptor-gamma delta T cell subsets during fetal ontogeny. J. Immunol. **141(11):** 3753–3759.

Ikuta K., Kina T., MacNeil I., Uchida N., Peault B. Chien Y.H., and Weissman I.L. (1990). A developmental switch in thymic lymphocyte maturation potential occurs at the level of hematopoietic stem cells. Cell **62(5):** 863–74.

Ito K., Bonneville M., Takagaki Y., Nakanishi N., Kanagawa O., Krecko E.G., and Tonagawa S. (1989). Different $\gamma\delta$ T-cell receptors are expressed on thymocytes at different stages of development. Proc. Natl. Acad. Sci. USA **86:** 631–635.

Itohara S. Farr A.G., Lafaille J.J., Bonneville M., Takagaki Y., Haas W., and Tonegawa S. (1990). Homing of a $\gamma\delta$ thymocyte subset with homogeneous T-cell receptors to mucosal epithelial. Nature **343:** 754–757.

Itohara S., Mombaerts P., Lafaille J.J., Iacomini J., Nelson A., Clarke A.R., Hooper M.L., Farr A.G., and Tonegawa S. (1993). T cell receptor $\delta$ gene mutant mice: independent generation of $\alpha\beta$ T cells and programmed rearrangements of $\gamma\delta$ TCR genes. Cell **72(3):** 337–348.

Jotereau F., Heuze F., Salomon-Vie V., and Gascan H. (1987). Cell kinetics in the fetal mouse thymus: precursor cell input, proliferation, and emigration. J. Immunol. **138:** 1026–1030.

Kelly K.A., Pearse M., Lefrancois L., and Scollay R. (1993). Emigration of selected subsets of $\gamma\delta^+$ T cells from the adult murine thymus. Int. Immunol. **5(4):** 331–335.

Ledbetter J.A., and Herzenberg L.A. (1979). Xenogeneic monoclonal antibodies to mouse lymphoid differentiation antigens. Immunol.Rev. **47(63):** 63–90.

Nikolic-Zugic J. (1991). Phenotypic and functional stages in the intrathymic development of $\alpha\beta$ T cells. Immunol. Today **12(2):** 65–70.

Ogimoto M., Yoshikai Y., Matsuzaki G., Matsumoto K., Kishihara K., and Nomoto K. (1990). Expression of T cell receptor V gamma 5 in the adult thymus of irradiated mice after transplantation with fetal liver cells. Eur. J. Immunol. **20(9):** 1965–1970.

Penit C., and Vasseur F. (1989). Cell proliferation and differentiation in the fetal and early postnatal mouse thymus. J. Immunol. **142:** 3369–3377.

Rothenberg E.V. (1992). The development of functionally responsive T cells. Adv. Immunol. **51:** 85–214.

Scollay R., and Shortman K. (1983). Thymocyte subpopulations: an experimental review, including flow cytometric cross-correlations between the major murine thymocyte markers. Thymus **5:** 245–295.

Shortman K. (1992). Cellular aspects of early T-cell development. Curr. Opin. Immunol. **4(2):** 140–146.

Shortman K., Williams N., and Adams P. (1972). The separation of different cell classes from lymphoid organs. V. Simple procedures for the removal of cell debris. Damaged cells and erythroid cells from lymphoid cell suspensions. J. Immunol. Methods **1(3):** 273–287.

Springer T., Galfre G., Secher D.S., and Milstein C. (1978). Monoclonal xenogeneic antibodies to murine cell surface antigens: identification of novel leukocyte differentiation antigens. Eur. J. Immunol. **8(8):** 539–551.

Wilson A., D'Amico A., Ewing T., Scollay R., and Shortman K. (1988). Subpopulations of early thymocytes. A cross-correlation flow cytometric analysis of adult mouse $Ly\text{-}2^-L3T4^-(CD8^-CD4^-)$ thymocytes using eight different surface markers. J. Immunol. **140(5):** 146–1469.

Zorbas M., and Scollay R. (1993). Development of $\gamma\delta$ T cells in the adult murine thymus. Eur. J. Immunol. **23:** 1655–1660.

